# Correction

**DOI:** 10.1080/14756366.2023.2297117

**Published:** 2023-12-21

**Article title:** Design, Synthesis and Biological Investigation of Selective Human Carbonic Anhydrase II, IX and XII Inhibitors Using 7-Aryl/Heteroaryl Triazolopyrimidines Bearing a Sulfanilamide Scaffold

**Authors:** Romeo Romagnoli, Tiziano De Ventura, Stefano Manfredini, Erika Baldini, Claudiu T. Supuran, Alessio Nocentini, Andrea Brancale, Roberta Bortolozzi, Lorenzo Manfreda, Giampietro Viola

**Journal:** *Journal of Enzyme Inhibition and Medicinal Chemistry*

**Bibliometrics:** Volume 38, Number 1

**DOI:** https://doi.org/10.1080/14756366.2023.2270180

When the article has been previously published, **Dr Carmine Varricchio’s** name has not been included.

Below is the correct Author List for this article:

1. Romeo Romagnoli
2. Tiziano De Ventura
3. Stefano Manfredini
4. Erika Baldini
5. Claudiu T. Supuran
6. Alessio Nocentini
7. Andrea Brancale
8. **Carmine Varricchio -** School of Pharmacy and Pharmaceutical Sciences, Cardiff University, Cardiff, UK
9. Roberta Bortolozzi
10. Lorenzo Manfreda
11. Giampietro Viola

